# Investigations and treatment offered to women presenting for infertility care in Harare, Zimbabwe: a cross sectional study

**DOI:** 10.11604/pamj.2021.40.191.27928

**Published:** 2021-11-30

**Authors:** Mugove Gerald Madziyire, Thulani Lesley Magwali, Vasco Chikwasha, Tinovimba Mhlanga

**Affiliations:** 1 Department of Obstetrics and Gynaecology, University of Zimbabwe College of Health Sciences, Harare, Zimbabwe,; 2 Department of Community Medicine, University of Zimbabwe College of Health Sciences, Harare, Zimbabwe

**Keywords:** Infertility investigations, subfertility investigations, infertility treatments

## Abstract

**Introduction:**

clinical infertility is failure to conceive within a year of regular sexual intercourse by a non contracepting couple. Infertility care is costly and result in investigations being incomplete and inconclusive. It is therefore important to streamline investigations offered to infertile couples such that only the most cost effective tests are done. This paper explores the adequacy of investigations and treatments offered to women presenting for infertility care.

**Methods:**

the data used in this analysis was obtained from a cross sectional sample of 216 women who presented with infertility in public and private gynaecological clinics in Harare, Zimbabwe. Information on investigations and treatment offered to women presenting for care was extracted from hospital cards, case notes, laboratory and radiological reports. Data was analysed using STATA SE/15.

**Results:**

of the 178 (82.4%) who had ultrasound scan evaluation (USS) 50 (28.1%) had fibroids and 22 (12.4%) had polycystic ovaries. Tubal patency tests were done in 118 participants using (hystero-salpingogram) HSG alone in 62.7%, laparoscope and dye alone in 21.2% and both in 16.1% of them. Of the 97 (44.9%) men who had semen analysis 61 (62.9%) had abnormal parameters.

**Conclusion:**

this study reveals that evaluation for tubal patency and USS to rule out reproductive organ pathology are not being offered to all women with a diagnosis of infertility. Likewise, male partner semen evaluation is not being done in all male partners. There is a high prevalence of abnormal semen parameters. Studies should be done to understand why some male partners are not forthcoming in providing semen for analysis. It is important for protocols to be produced by professional bodies which prescribe the minimum basic investigations in couples with infertility.

## Introduction

Clinical infertility is failure to conceive within a year of regular sexual intercourse by a non contracepting couple [1]. It is a common presentation in gynaecology clinics, accounting for up to 20% of women coming for consultation [2]. The International conference on population and development (ICPD) Cairo conference recognised it as a priority reproductive health area [3]. Furthermore reproductive rights were expounded as the free will of couples or individuals to have a right to decide on the number, spacing, and timing of their children, and the information and means to do so [4]. World Health Organization (WHO) sustained development goals (SDG) target 3.7 speaks to the need to ensure universal access to sexual and reproductive health, including infertility prevention and treatment [5]. World Health Organization advocates for a strong response to the unmet met need for infertility care services; development of infertility diagnosis and care guidelines and research to answer priority questions [6].

Infertility treatment is not a priority area for most governments in low and middle income countries, medical insurance companies and funding partners whose priority is to address issues of high maternal mortality and overpopulation. Consequently, individuals or couples have to rely on their own resources to fund costly investigations and treatment. This is compounded by the fact that infertility care is costly and result in investigations being incomplete and inconclusive. Hence there is limited access to infertility care in the developing world [7]. Consequently, the number of patients with a conclusive diagnosis and those who achieve conception is presumed to be low. It is therefore important to streamline investigations offered to infertile couples such that only the most cost-effective tests are done. No study has analysed the adequacy, pattern of investigations and treatments offered to women presenting with infertility in gynaecology clinics in Zimbabwe. The findings will inform management of infertile couples in resource constrained set-ups. This paper hence explores the investigations and treatment offered to women presenting with infertility in gynaecology clinics in Harare, Zimbabwe during the study period.

## Methods

The data used in this analysis was obtained from a cross sectional sample of 216 women who had presented with infertility in public and private gynaecological clinics in Harare, Zimbabwe. The study population and sampling has been reported in another paper [8].

Information on investigations offered to women presenting for care was extracted from hospital cards, case notes, laboratory and radiological reports. Serum progesterone was assessed for evidence of ovulation while luteinising hormone (LH), follicle stimulating hormone (FSH) and anti-mullerian hormone (AMH) were assessed for ovarian reserve. Testosterone was assessed for evidence of hyper-androgenism. Semen analysis was assessed for various degrees of sperm dysfunction using WHO 2010 classification [9] while hysterosalpingogram (HSG) and laparoscope and dye studies (lap and dye) where used to evaluate tubal patency.

Data was collected using a questionnaire and captured into Redcap software [10] and exported to STATA/SE 15.1 for analysis. Tests for association were conducted using the chi-square test for categorical variables. Where sample size was small in some cells the Fisher´s exact test was used.

**Ethics approval and consent to participate:** ethical clearance was granted by the Joint Research Ethics Committee for the University of Zimbabwe, College of Health Sciences and Parirenyatwa Group of Hospitals (JREC) on 8^th^ May 2019, Harare hospital ethics committee on 15^th^ April 2019, Chitungwiza hospital ethics committee on 15^th^ April 2019 and the Medical Research Council of Zimbabwe (MRCZ) on 20 May 2019.

## Results

**Figure 1:**
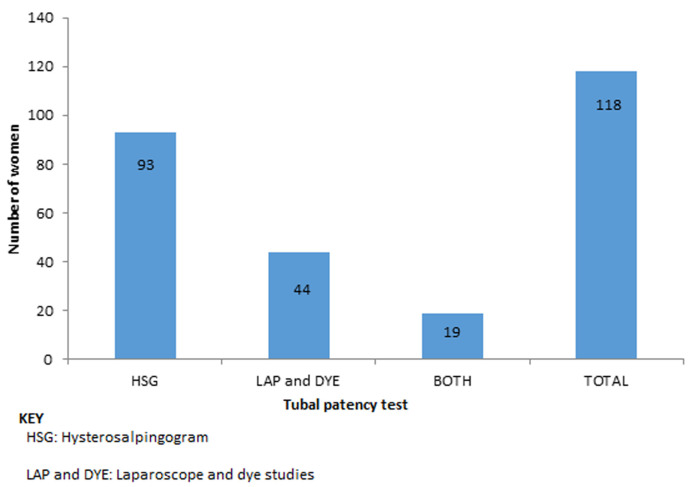
tubal patency tests

Data was analysed from 216 participants of which 47 (22%) were from the public sector while 169 (78%) were from the private sector. The demographics, reproductive characteristics and diagnosis of this population has been reported in another paper [8]. Of the 178 (82.4%) who had ultrasound scan (USS) examinations, almost half of them (49%) had normal findings while 50 (28.1%) had fibroids and 22 (12.4%) had polycystic ovaries. Tubal patency tests were done in 118 (54.6%) participants using HSG alone in 62.7%, Lap and dye alone in 21.2% and both in 16.1% of them. Of the 93 (43.1%) who had HSG examination 39 (42%) of them had bilateral tubal blockage while of the 44 (21.4%) who had lap and dye examination 15 (34%) had bilateral tubal blockage (Figure 1).

Half of the 68 participants who had a progesterone assay had a normal level supportive of an ovulatory cycle. Of the 90 participants who had LH assessment and 105 who had FSH, 11 (12%) and 6 (5.7%) of them respectively had high levels indicative of reduced ovarian reserve. Eleven (21.2%) of the 105 participants who had AMH measured had a low level indicative of reduced ovarian reserve. Only 7 (13%) of the 54 patients who had testosterone measured had a value supporting hyper-androgenism. Of the 65 participants who had thyroid-stimulating hormone (TSH) assessment 13 (20%) had elevated levels while out of the 81 who had prolactin measurement 11 (13.6%) had hyperprolactinaemia (Table 1). There was no association between serum progesterone (p=0.083), AMH (p=0.688) and FSH (p=0.11) level with menstrual cycle regularity. The mean age for women with AMH <1.2ng/ml was 37.7 years and this was significantly different in women who had AMH ≥1.2ng/ml (p=0.011).

**Table 1:** hormonal tests n (%)

	Not done	Normal	High	Low	Total done
**progesterone**	148(68.5)	34(15.7)		34(15.7)	68(31.5)
**LH**	126(58.3)	75(34.7)	11(5.1)	4(1.9)	90(41.7)
**FSH**	111(51.4)	74(34.3)	6(2.8)	25(11.6)	105(48.6)
**AMH**	164(75.9)	41(19)		11(5.1)	52(24.1)
**Testosterone**	162(75)	36(16.7)	7(3.2)	11(5.1)	54(25)
**TSH**	151(69.9)	50(23.2)	13(6)	2(0.9)	65(30.1)
**prolactin**	135(62.5)	67(31)	11(5.10	3(1.4)	81(37.5)

Progesterone - ≥5ng/ml was taken as normal since it indicates ovulation in the luteal phase. Low: <5ng/ml; LH: luteinising hormone. Normal: 2-15iu/l; High >15iu/l; low <2iu/l; FSH: follicle Stimulating hormone: Normal 3-20iu/l, high >20iu/l; low<3iu/l; AMH: Anti-mullerian hormone: normal/good response ≥1.2ng/ml; low or poor response <1.2ng/ml (posseidon classification); Testosterone: normal: 0.5-2.5nmol/l(0.144-0.72ng/ml); high; >2.5nmol/l or >0.72ng/ml; low <0.5nmol/l or <2.5nmol/l; TSH: Thyroid stimulating hormone: normal: 0.35-5iu/ml; high: 5iu/ml; low: <0.35iu/ml; Prolactin: normal: 5-25ng/dl or 100-500miu/l; high: >25ng/dl/>500miu/l; low: < 5ng/dl/100miu/l

**Figure 2:**
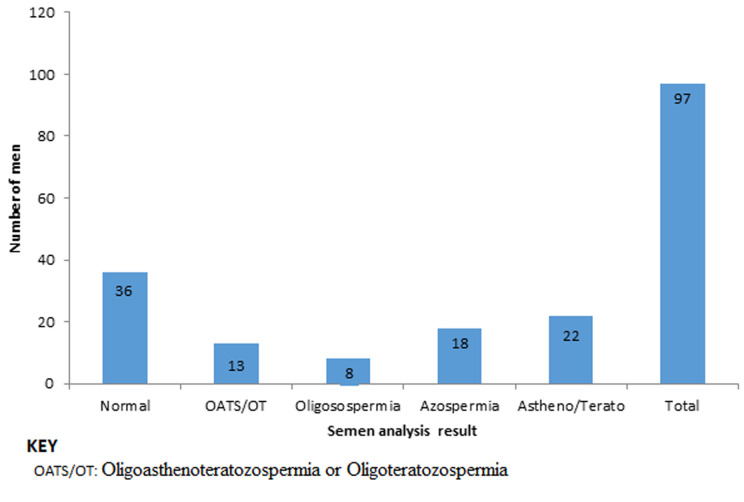
results of semen analysis

Sperm analysis was done in 97 (44.9%) of the participants and 39 (40%) of them had varying degrees of oligozospermia. Sperm morphology and motility was recorded in 80 and 90 of the participants´ records respectively. Of the men who had sperm analysis 61 (62.9%) had abnormal parameters with 18 (18.6%) with azoospermia and 13 (13.4%) with Oligoasthenoteratozospermia or Oligoteratozospermia (OATS/OT) (Figure 2). Seventy-six (64.8%) had ovulation induction with most of them (51 (67%) getting clomiphene or letrozole. Thirty-seven (17.1%) women had antiretroviral therapy (ART) with most of them (22) getting IVF/ICSI in Zimbabwe. Of the 36 women who were offered surgical interventions, the most common procedure was myomectomy 15 (42%). Women with a diagnosis of anovulation were significantly more likely to have ovulation induction than those with other diagnosis (x^2^=9.89, p=0.002).

## Discussion

Ultra-sound scan was the most common radiological investigation done and as expected fibroids were the commonest lesion to be found. Ultra-sound scan should be offered to all women presenting with infertility. It allows assessment of pelvic organs, excluding anomalies while showing physiological changes [11]. The prevalence of fibroids in this population (28%) is higher than that 5-10% quoted in one review [12]. Assessment for the presence, size and location of fibroids remains an invaluable exercise in infertility treatment. While most fibroids don´t cause infertility, removal of big fibroids or submucous fibroid which disturb the uterine geometry or indent on the endometrium or uterine cornua can improve chances of fertility [13]. It is important that submucous fibroids are assessed using transvaginal scan (TVS) so as to improve accuracy of the evaluation. Just over half of the participants had tubal patency tests with HSG being the predominant procedure. This a cost-effective procedure for establishing tubal patency and should be offered ahead of lap and dye in women without pelvic pain [14]. Tubal patency evaluation is essential and must be offered universally to all women with infertility. It is highly imperative that all units attending to infertility patients have a readily accessible cost-effective way of establishing tubal patency. Sonohysterogram is an alternative procedure which can be performed as an office procedure and has better sensitivity and specificity than HSG [15]. It is commendable that practitioners relied less on Laparoscope and dye as it is more invasive and costly. However, it is essential when there is pelvic pain and allows practitioners to rule out pelvic pathology [16].

Hormonal tests were done on less than half the clients. These tests have a crucial role in giving evidence of ovulation and ovarian reserve. They are costly and hence the tendency for them to be offered only where there is clinical evidence of anovulation such as irregular, infrequent or absent menses or in advanced maternal age. There was no statistically significant association between cycle regularity and serum progesterone level. This could be due to the fact that some practitioners were ordering it outside the luteal phase. Ideally serum progesterone should be done about 7 days before the next expected menses [17]. Luteal phase progesterone was commonly used to predict ovulation. This is invasive and costly. Clinicians must be advised to consider alternatives which are less invasive and cheaper such as urinary LH kits which detect the LH surge prior to ovulation. Basal body temperature which picks the thermogenic response to increased serum progesterone by the corpus luteum after ovulation is not invasive and is cheap notwithstanding the cumbersome routine of daily temperature measurements [18]. Low AMH was significantly associated with advanced maternal age unlike FSH and LH. This is likely because AMH measures ovarian function throughout the menstrual cycles whereas LH and FSH can only be measured around day 3 and this might not have been timed correctly in some cases. Anti-mullerian hormone, maternal age and antral follicle count (Poseidon classification) are considered the most reliable in predicting ovarian reserve [19]. Majority (62%) of the men who had sperm analysis had some abnormality. Other studies have also shown high prevalence sperm dysfunction in infertile couples. Studies in Saudi Arabia found prevalence of a single abnormality of 52.7% and 65.6% respectively in male partners of infertile couples [20,21].

Another study in Abakaliki, Nigeria found a prevalence of highly defective semen parameters of 64% [22]. This high prevalence of abnormal semen parameters in infertile couples can be explained by the fact that WHO reference ranges were set using the 5th percentile of a population of men with normal fertility [9]. It is important therefore to evaluate more male partners in couples presenting with infertility as lesser degrees of sperm dysfunction can be managed using Intrauterine insemination (IUI) with washed sperms. It is commendable that ovulation induction was likely to be given to women with a diagnosis of anovulation rather than empirically as a trial. Ovulation inductions with antioestrogens such as clomiphene citrate or tamoxifen or with an aromatase inhibitor such as letrozole improves pregnancy rates in women with conditions that cause anovulation [23]. It is debatable whether a trial of ovulation induction is beneficial in women without evidence of anovulation. A Cochrane review found increased pregnancy rates when clomiphene citrate was given to women with unexplained infertility [24]. This study provides the first ever evaluation of investigations and treatments offered to couples presenting with infertility. Its weakness is that it collates data from participants seen in different units who are not following a common protocol. The analysis could not tell whether certain essential investigations were not done because of financial reasons or caregiver oversight. It however brings out a pattern of investigating and treating infertile couple which is peculiar to an urban setting in a developing country.

## Conclusion

This study reveals that evaluation for tubal patency and USS to rule out reproductive organ pathology are not being offered to all women with a diagnosis of infertility. Likewise, male partner semen evaluation is not being done in all male partners. There is a high prevalence of abnormal semen parameters. Studies should be done to understand why some male partners are not forthcoming in providing semen for analysis. It is important for protocols to be produced by professional bodies which prescribe the minimum basic investigations in couples with infertility.

**Funding:** research reported in this publication was supported by the Fogarty International Center (Office Of The Director, National Institutes Of Health (OD), National Institute Of Nursing Research (NINR), National Institute Of Mental Health (NIMH), National Institute Of Dental and Craniofacial Research (NIDCR), National Institute Of Neurological Disorders And Stroke (NINDS), National Heart, Lung, And Blood Institute (NHLBI), Fogarty International Center (FIC)) of the National Institutes of Health under Award Number D43 TW010137. The content is solely the responsibility of the authors and does not necessarily represent the official views of the National Institutes of Health.

### What is known about this topic

- Tubal patency, semen analysis, pelvic organs ultrasound assessment and ovulation must be assessed in every couple with subfertility.

### What this study adds

- There is incomplete evaluation of couples for tubal patency;
- Evaluation of pelvic pathology and function using ultrasound scan is not being offered to all women presenting with infertility;
- There is a high prevalence of abnormal semen parameters in couples presenting with infertility in Zimbabwe.
